# Chronobiome medicine: circadian regulation of host–microbiota crosstalk in systemic physiology

**DOI:** 10.3389/fendo.2025.1691172

**Published:** 2025-11-04

**Authors:** Jhommara Bautista, Andrés López-Cortés

**Affiliations:** Cancer Research Group (CRG), Faculty of Medicine, Universidad de Las Américas, Quito, Ecuador

**Keywords:** circadian rhythms, peripheral clocks, gut microbiota, metabolite production, circadian-microbiota axis, therapeutic strategies

## Abstract

Circadian rhythms, governed by central and peripheral clocks, orchestrate nearly all aspects of human physiology, including metabolism, endocrine function, neuroimmune activity, and behavior. Emerging evidence reveals that these oscillations are closely intertwined with the gut microbiota, which itself displays diurnal fluctuations in composition and metabolite production. This bidirectional regulation establishes a dynamic circadian–microbiota axis that synchronizes nutrient processing, hormonal secretion, immune surveillance, and neural signaling. Disruption of this temporal alignment, through genetic, environmental, or lifestyle factors, precipitates systemic dysregulation, fostering metabolic syndrome, endocrine imbalance, immune dysfunction, neuropsychiatric vulnerability, cardiovascular alterations, and carcinogenesis. Mechanistic studies highlight that microbial-derived metabolites such as short-chain fatty acids, bile acids, and indoles act as circadian cues, while host clock genes modulate microbial ecology and intestinal barrier integrity. These insights underscore the translational potential of circadian precision medicine, in which time-restricted feeding, probiotics, prebiotics, and chronotherapy restore synchrony between microbial and host clocks. This review synthesizes current knowledge on circadian modulation of microbiota-mediated crosstalk across metabolic, neural, immune, and endocrine pathways, emphasizing its implications for health, disease, and novel therapeutic strategies.

## Introduction

**Figure 1:**
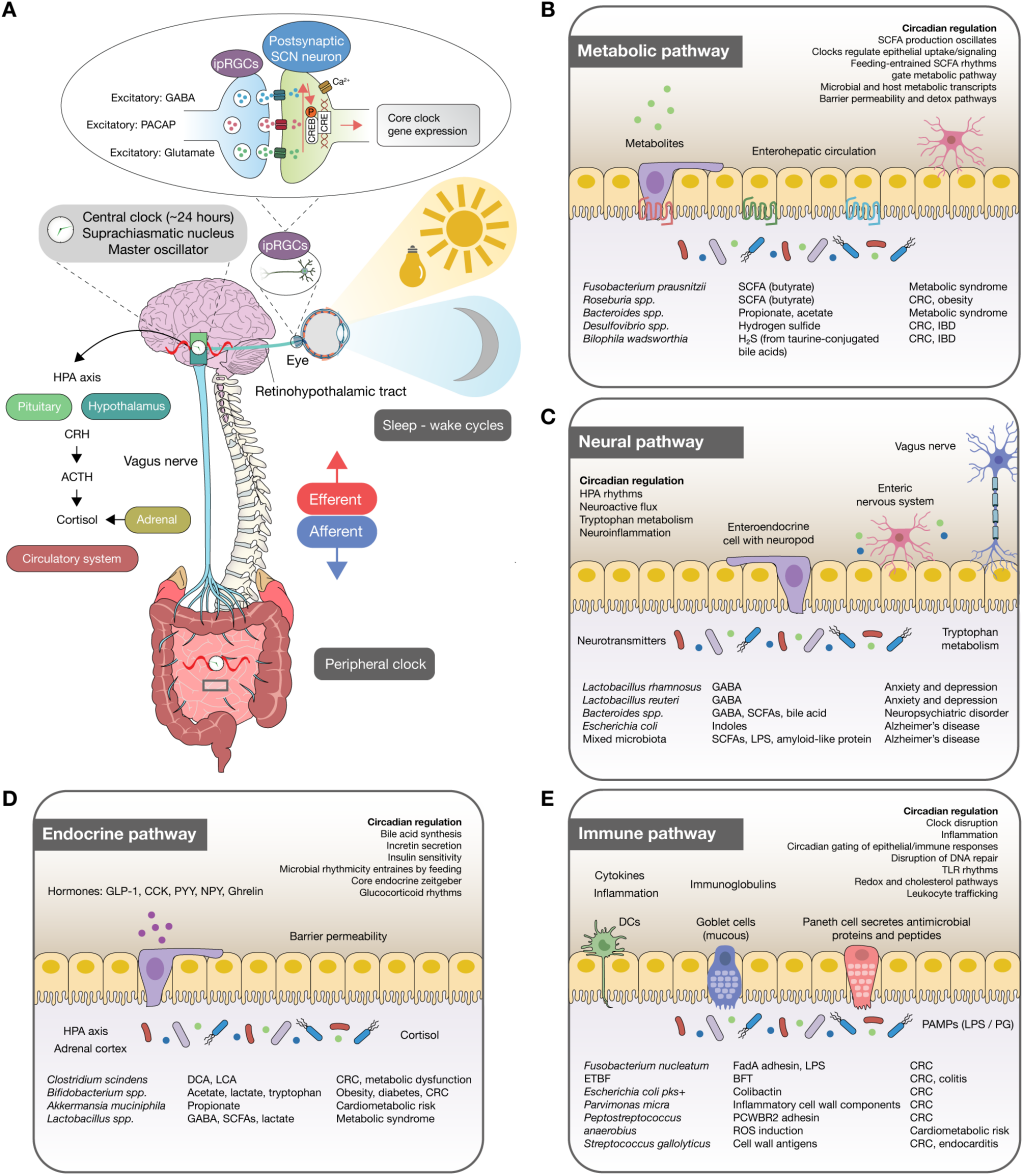
Circadian control of the gut–brain axis linking microbiota to disease across immune, metabolic, neural, and endocrine pathways. **(A)** Light cues synchronize intrinsically ipRGCs, which signal to the suprachiasmatic nucleus (SCN; master circadian oscillator) via glutamate, PACAP, and GABA to drive core clock gene expression. SCN outputs align peripheral clocks through autonomic (vagus) and humoral routes (HPA axis) and by sleep–wake timing. Efferent and afferent pathways couple the brain and intestine, entraining epithelial, immune, neuronal, and endocrine programs. **(B)** Immune pathway. The intestinal epithelium, goblet cells, Paneth cells, and dendritic cells sense microbial PAMPs. Local clocks gate epithelial/immune responsiveness, TLR rhythms, leukocyte trafficking, redox/cholesterol pathways, DNA repair, and inflammation. Circadian disruption amplifies ROS and pro-inflammatory tone, contributing to cardiometabolic risk. **(C)** Metabolic pathway. Feeding-entrained rhythms regulate SCFA production and epithelial uptake/signaling, coordinate microbial/host metabolic transcripts, and influence barrier permeability and detoxification. **(D)** Neural pathway. Vagal and enteric nervous system circuits receive time-gated microbial signals and enteroendocrine “neuropod” outputs. Clocks modulate HPA rhythms, neuroactive flux, tryptophan/kynurenine metabolism, and neuroinflammation. **(E)** Endocrine pathway. Circadian clocks control bile-acid synthesis, incretin release (GLP-1, CCK, PYY, NPY, ghrelin), and insulin sensitivity; microbial rhythmicity is entrained by feeding and feeds back to endocrine clocks. HPA-axis glucocorticoids alter epithelial barrier function. ipRGCs, intrinsically photosensitive retinal ganglion cells; PACAP, pituitary adenylate cyclase–activating polypeptide; GABA, γ-aminobutyric acid; SCN, suprachiasmatic nucleus; HPA, hypothalamic–pituitary–adrenal; PAMPs, pathogen-associated molecular patterns; PG, peptidoglycan; TLR, Toll-like receptor; SCFAs, short-chain fatty acids.

Circadian rhythms are endogenous oscillations with an approximately 24-hour periodicity that regulate virtually every aspect of physiology, ranging from sleep-wake cycles and hormonal secretion to immune responses and metabolism (1). These rhythms are orchestrated by a central pacemaker localized in the suprachiasmatic nucleus (SCN) and by autonomous peripheral clocks distributed across nearly all organ systems, including the liver, intestine, adipose tissue, pancreas, and immune cells (2). At the molecular level, circadian rhythms are generated by transcriptional–translational feedback loops involving core clock genes such as *CLOCK*, *BMAL1*, *PER*, and *CRY*, whose rhythmic expression synchronizes physiology with both environmental and internal *zeitgebers*, including light, feeding patterns, temperature, and hormonal fluctuations (3) (**Figure 1A**). Importantly, emerging evidence indicates that the circadian system is intricately intertwined with the human microbiota, forming a bidirectional regulatory axis with profound implications for health and disease (4–6).

Disruption of circadian rhythms, whether caused by genetic alterations, shift work, or lifestyle misalignment, leads to systemic desynchronization, strongly associated with metabolic dysfunction, immune dysregulation, neurodegeneration, and cancer (3, 7–9). At the metabolic level, peripheral clocks in tissues such as the liver, muscle, and adipose tissue synchronize nutrient processing with anticipated feeding times, optimizing digestion, absorption, and energy storage (2). Circadian misalignment compromises this temporal organization, driving insulin resistance, obesity, and type 2 diabetes (10). The gut microbiota also exhibits diurnal oscillations regulated in part by the intestinal epithelial cell clock, contributing to metabolic homeostasis through the production of short-chain fatty acids (SCFAs) and bile acids that act as systemic metabolic cues (11). A seminal study by Thaiss et al. demonstrated that microbial communities oscillate in abundance and function in synchrony with host feeding rhythms, and that perturbation of these rhythms impairs metabolic health (12). Disruption of the intestinal clock alters microbial rhythmicity, leading to dysbiosis, impaired barrier function, and increased susceptibility to cancer development (13). Notably, diurnal microbial programs are blunted by mistimed feeding and high-fat diets, which dampen community oscillations and remodel metabolite rhythms linked to host glucose-lipid homeostasis (14–16).

The endocrine system represents a critical interface between circadian and microbial regulation. Hormones such as cortisol, melatonin, insulin, leptin, ghrelin, and sex steroids exhibit robust daily oscillations that not only respond to circadian control but also serve as *zeitgebers* for peripheral clocks (17). Conversely, gut microbial metabolites, including SCFAs and secondary bile acids, influence hormone secretion and sensitivity, reinforcing a feedback loop between circadian oscillations and endocrine homeostasis (5, 18, 19). Disruption of this reciprocal regulation perturbs neuroendocrine signaling and metabolic balance, predisposing individuals to disorders ranging from metabolic syndrome to hormone-dependent cancers (17). Supporting evidence from human and animal studies show that feeding-time shifts rephase bile acid and SCFA rhythms, altering incretin signaling and insulin sensitivity (14, 15).

Immune function is likewise under strong circadian control. Core clock genes modulate the maturation, trafficking, and effector activity of immune cells, while rhythmic cytokine secretion coordinates temporal immune surveillance. Circadian disruption alters immune homeostasis, promotes chronic inflammation, and reprograms the tumor microenvironment (TME) toward an immunosuppressive state (13, 20). Notably, microbial rhythms intersect with these immune dynamics. A striking example by Brooks et al. demonstrated that rhythmic microbial adhesion by segmented filamentous bacteria drives oscillations in epithelial STAT3–3 innate lymphoid cells (ILC3)–antimicrobial protein circuits, aligning mucosal immune defense with feeding rhythms (21). Clock disruption–induced dysbiosis contributes to aberrant cytokine release, defective barrier integrity, and tumor progression (6, 7). Mechanistically, the microbiota can set innate immune timing: daily segmented filamentous bacteria (SFB) attachment entrains epithelial–ILC3 circuits and antimicrobial peptide production, conferring time-of-day variation in pathogen resistance (21).

The gut-brain axis further exemplifies the integrative nature of circadian and microbial regulation. The microbiota modulates stress responsivity and neuroendocrine signaling through circadian-dependent regulation of the hypothalamic-pituitary-adrenal (HPA) axis (7, 22). Microbial depletion disrupts corticosterone rhythmicity, alters hippocampal and amygdalar stress pathways, and impairs daily stress adaptation. Conversely, transplantation of specific microbial taxa restores circadian regulation of stress hormones, underscoring the role of microbial diurnal oscillations in maintaining neuroimmune balance (23). In particular, transplantation or supplementation with defined strains, such as *Lactobacillus reuteri*, has been shown to re-entrain diurnal glucocorticoid rhythms and normalize time-of-day HPA-axis responses; complementary evidence implicates *Bifidobacterium longum* 1714 and *Lactobacillus rhamnosus* JB-1 in attenuating stress-evoked cortisol/corticosterone outputs, highlighting strain-level contributions to endocrine rhythmicity (24, 25). This interplay among microbiota, circadian clocks, and neuroendocrine circuits is increasingly recognized as a determinant of susceptibility to psychiatric and neurological disorders (26, 27). Translational studies further suggest that aligning feeding and light schedules restores microbial and endocrine rhythmicity, improving sleep quality and stress resilience (14, 21).

Importantly, specific microbiota parameters are consistently altered by host circadian disruption. Key changes include the loss or dampening of diurnal oscillations in microbial taxa and functions, blunted SCFA and bile acid rhythms, shifts in community structure (α/β-diversity) with enrichment of pathobionts, altered spatial ecology (e.g., flattened rhythmic mucosal adherence of SFB), metabolic output reprogramming (tryptophan-kynurenine pathway, indole derivatives, β-glucuronidase activity), and barrier-immune interface defects (mucus integrity, epithelial PRR tone, IgA coating). Major disruptors producing these signatures include shift work and social *jet lag*, mistimed feeding (14–16), circadian desynchrony caused by *jet lag* that rephases microbial bile acids and induces immunosuppressive myeloid programs (28), as well as light-at-night exposure and sleep restriction (21).

The pathological consequences of circadian–microbiota misalignment are particularly evident in cancer biology. Circadian clocks regulate cell proliferation, DNA repair, and apoptosis, and their disruption reprograms tumor suppressor and oncogene networks (9). Dysbiosis induced by circadian disruption further accelerates tumorigenesis through barrier dysfunction, genotoxic metabolites, and chronic inflammation. In colorectal cancer, circadian misalignment, induced by factors such as shift work, irregular feeding schedules, chronic *jet lag*, or light-at-night exposure, exacerbates dysbiosis and epithelial permeability, promoting microbial-driven oncogenic signaling. Transcriptomic profiling has identified circadian rhythm–dependent molecular subtypes of colorectal tumors, associated with distinct immune landscapes and therapeutic responsiveness (29). Recent mechanistic work links circadian desynchrony to metastatic progression via microbiota-driven taurocholic-acid signaling that expands immunosuppressive myeloid cells and dampens CD8^+^ T cell cytotoxicity (28, 30, 31).

This review synthesizes emerging knowledge on circadian regulation of the microbiota and its systemic implications. We discuss the molecular basis of circadian clocks and their influence on microbial rhythmicity; the reciprocal role of the microbiota in shaping circadian-controlled metabolism, neuroendocrine function, and immunity; and the pathological outcomes of circadian–microbiota misalignment, with emphasis on metabolic dysfunction, tumorigenesis, and stress-related disorders. Finally, we highlight the therapeutic promise of chronobiome medicine, which aims to harness microbiota-mediated crosstalk for clinical benefit.

## Circadian regulation of microbiota–metabolic axis

The circadian clock represents a fundamental biological system that orchestrates the temporal alignment of metabolic processes with daily environmental cycles, and its interactions with the gut microbiota constitute a critical axis for maintaining energy balance and systemic homeostasis (**Figure 1B**). The molecular machinery of circadian rhythms is embedded in nearly every nucleated cell and is driven by transcriptional–translational feedback loops (TTFLs) that generate oscillations in clock gene expression and their downstream targets (32). These oscillations regulate diverse metabolic pathways in peripheral tissues such as liver, adipose, muscle, and pancreas, ensuring that nutrient absorption, storage, and energy utilization occur at the most advantageous times of day (3, 33). Importantly, the circadian system does not act in isolation: it is intimately intertwined with feeding–fasting cycles and with the gut microbiota, whose composition and metabolic functions fluctuate rhythmically in parallel with host physiology (34, 35).

Importantly, the host molecular clock exerts direct control over microbial rhythmicity through both behavioral and physiological outputs. Feeding–fasting cycles regulated by the SCN generate rhythmic nutrient availability that entrains microbial growth and metabolism, while peripheral clocks, particularly in intestinal epithelial cells, govern barrier permeability, mucus secretion, and bile acid transport, thereby structuring the temporal niches that microbes occupy (12, 16). Loss of epithelial clock components such as BMAL1 disrupts microbial oscillations and metabolite rhythmicity, highlighting a causal role for host timing mechanisms in shaping microbial ecology (5). Moreover, the transcription factor NFIL3, whose expression is co-regulated by the circadian clock and microbial signals, synchronizes intestinal lipid absorption and immune gene expression, linking clock-driven epithelial programs to microbial composition and metabolic function (36) Collectively, these mechanisms demonstrate that the host clock not only responds to microbial cues but also actively entrains microbial rhythmicity through coordinated behavioral, metabolic, and epithelial outputs.

Disruption of circadian rhythms, through shift work, irregular sleep, or mistimed feeding, has profound consequences on host metabolism. Epidemiological and experimental studies consistently link circadian misalignment to obesity, insulin resistance, type 2 diabetes, and cardiovascular disease (3, 10, 37). At the mechanistic level, disruption of circadian clocks alters the transcriptional regulation of key metabolic enzymes and hormone signaling pathways, thereby uncoupling metabolic demands from nutrient availability. Simultaneously, the gut microbiota, which relies on nutrient supply patterns dictated by host feeding rhythms, undergoes compositional and functional dysbiosis under circadian disruption (38). This dual misalignment amplifies metabolic stress, fueling chronic low-grade inflammation and metabolic syndrome (4, 39).

The gut microbiota is not merely a passive recipient of circadian cues but an active participant in regulating host metabolic rhythms. In mice, fecal SCFAs show a clear diurnal peak ~4 hours after lights-on (ZT4) and lose rhythmicity in Bmal1^−^/^−^ mice—an effect that can be restored by aligning feeding time; functionally, cecal SCFA concentrations inhibit colonic ghrelin release at ZT4 but not at ZT16 (40, 41). In humans, fecal acetate, propionate, and butyrate are highest in the morning and decline toward evening, indicating conserved day–night SCFA dynamics (42). Microbial fermentation products therefore act as metabolic time cues that influence host clock gene expression, nutrient sensing, and energy metabolism, and these effects are blunted by circadian disruption or high-fat diets (15, 16, 43).

Time-restricted eating (TRE) exemplifies how synchronizing feeding schedules with circadian biology can restore metabolic health. TRE realigns peripheral clocks by providing temporally restricted nutrient inputs that entrain gut microbial rhythms, thereby enhancing rhythmicity in SCFA production, bile acid metabolism, and glucose–lipid pathways (5). Omics analyses reveal that TRE reshapes transcriptional programs of both circadian genes and metabolic regulators, leading to improved insulin sensitivity, reduced adiposity, and better lipid profiles (44–46). The microbial rhythms modulate host epigenetic regulation, nuclear receptor signaling, and hormone secretion, effectively acting as mediators of circadian–metabolic benefits.

Importantly, the circadian regulation of the microbiota–metabolic axis is not uniform but depends on dietary composition and environmental cues. High-fat, Western-style diets blunt microbial rhythmicity, shifting the balance toward pathobionts and reducing oscillations in beneficial taxa such as *Akkermansia* and *Faecalibacterium* (19, 47). This disruption dampens SCFA production and enhances pro-inflammatory metabolites, thereby weakening circadian coherence between host and microbiota. Conversely, diets rich in fiber and prebiotics amplify microbial rhythmicity, supporting stable circadian–metabolic coupling (47, 48). Furthermore, bile acid signaling emerges as a key mediator: microbiota-driven bile acid transformations exhibit circadian oscillations that influence lipid absorption, glucose tolerance, and enterohepatic signaling, linking microbial activity to metabolic homeostasis (19).

At the systemic level, daily rhythms integrate microbial signals with host hormonal axes. For instance, microbial-derived metabolites modulate the secretion of incretins, leptin, and ghrelin, thereby influencing satiety and feeding behavior (18, 49). Melatonin, traditionally viewed as a pineal hormone, is also produced in the gut and metabolized by microbes; this local melatonin–microbiota axis feeds back on host circadian networks and impacts metabolic outcomes, highlighting novel therapeutic targets for rhythm-associated disorders (19). Moreover, disruption of sleep, a hallmark of circadian dysregulation, exacerbates microbial and metabolic disturbances, as evidenced in human cohorts where chronic insomnia correlates with gut dysbiosis, altered bile acid metabolism, and increased cardiometabolic risk (39).

From a clinical perspective, circadian disruption-induced microbial dysbiosis contributes to the pathogenesis of obesity, diabetes, and metabolic syndrome (50). Human trials demonstrate that altering microbial energy harvest through diet composition modulates host energy balance, with interindividual variability explained by SCFA production and microbial biomass (47). Thus, the microbiota–circadian interface serves as both a biomarker of metabolic health and a therapeutic target. Precision strategies such as timed feeding, microbiota-targeted diets, probiotic supplementation, and possibly microbial melatonin modulation hold promise for re-establishing circadian–metabolic harmony (4, 19, 33).

Collectively, converging evidence from molecular, translational, and clinical studies underscores that circadian regulation of the microbiota–metabolic axis is fundamental for maintaining metabolic homeostasis. Perturbations of this axis, driven by lifestyle factors, dietary patterns, or genetic predisposition, disrupt the temporal synchrony between microbial and host clocks, thereby promoting metabolic dysfunction and disease. In contrast, strategies that re-establish circadian–microbial alignment, including time-restricted nutrition, targeted microbiome modulation, and chronotherapy, hold significant potential to restore systemic balance and mitigate disease risk. Thus, the microbiota–metabolic circadian axis emerges as a central paradigm in which temporal biology, microbial ecology, and metabolic physiology intersect to shape health outcomes across the human lifespan.

## Circadian regulation of microbiota–neural axis

The circadian system orchestrates temporal homeostasis across multiple physiological domains, with the SCN acting as the central pacemaker that synchronizes peripheral clocks, including those in the gut and brain. This synchronization is critical for maintaining coherent communication along the microbiota–neural axis, where microbial signals dynamically interact with host circadian pathways to regulate neuroendocrine, immune, and cognitive functions (23) (**Figure 1C**). Disruptions to this alignment are increasingly implicated in the onset and progression of neuropsychiatric and neurodegenerative conditions, highlighting the circadian–microbiome–neural interface as a pivotal determinant of brain health. Evidence from molecular, preclinical, and clinical studies demonstrates that circadian regulation shapes the oscillatory behavior of gut microbiota, which in turn influences neurotransmitter synthesis, HPA axis activity, neuroinflammatory responses, and cognitive performance (7, 51, 52).

Central to this regulation is the rhythmicity of gut microbiota composition and function. Microbial taxa such as *Lactobacillus* and *Bacteroides* exhibit time-of-day-dependent fluctuations in abundance and metabolic output, producing SCFAs, bile acids, and neuroactive compounds that act as *zeitgebers* for host circadian machinery (12, 23, 43, 53). These microbial oscillations feedback to modulate clock gene expression in neural circuits and peripheral tissues, thereby reinforcing circadian homeostasis. Experimental depletion of gut microbes or circadian misalignment leads to profound alterations in corticosterone rhythms, hippocampal transcriptomes, and amygdala stress pathways, establishing microbiota as a critical modulator of neural circadian rhythmicity (7). A recent study by Tofani et al. provides direct evidence that the microbiota regulate stress responsivity in a circadian fashion and are necessary for adaptive, time-of-day–dependent HPA axis responses (7).

The neural implications of this crosstalk are particularly evident in stress responsivity and emotional regulation. Studies demonstrate that microbial oscillations directly shape diurnal patterns of glucocorticoid secretion, thereby influencing HPA axis function and stress-adaptive behaviors (7, 54). When circadian–microbiota alignment is disrupted, glucocorticoid release becomes dysregulated, driving maladaptive stress responses and impairing resilience to environmental challenges (7). These processes are mediated by the proximity and interconnectedness of the SCN and the paraventricular nucleus of the hypothalamus, which integrate circadian and stress signals. Microbiota-targeted interventions, such as probiotic administration, restore rhythmicity in glucocorticoid release and reduce anxiety-like behaviors, offering translational potential for mood and stress-related disorders (55, 56).

Mechanistic insights further reveal that communication between microbiota and the nervous system occurs through multiple conduits. Immune signaling represents one critical pathway, as cytokines and microbial-associated molecular patterns influence neuroinflammation and glial function in circadian-dependent ways (24, 51, 57). Neural pathways, particularly vagal afferents, transmit microbial and metabolic cues directly to the brainstem and higher-order neural centers, linking peripheral circadian states to central processes. Endocrine mediators, including serotonin, melatonin, and SCFAs, further reinforce circadian regulation of brain activity (55). Collectively, these inter-organ signaling mechanisms substantiate the microbiota–neural axis as a key effector of circadian integration across immune, neural, and endocrine dimensions.

Clinical and translational research highlights the consequences of circadian disruption on the microbiota–neural axis in pathological contexts. Aging and neurodegenerative disorders such as Alzheimer’s disease (AD) exemplify the vulnerability of this system. Circadian deterioration in the elderly exacerbates oxidative stress and immune dysregulation, priming the brain for neurodegeneration (51). Concurrently, gut dysbiosis in AD has been linked to impaired circadian rhythmicity, suggesting a synergistic deterioration of microbiota and circadian integrity that accelerates disease progression (58). Indeed, patients with neurodegenerative conditions often present with profound sleep and circadian abnormalities, which not only serve as clinical hallmarks but also contribute causally to neuropathological cascades. Experimental models confirm that circadian misalignment aggravates amyloid-β pathology, synaptic dysfunction, and memory deficits, reinforcing the mechanistic role of microbiota–circadian–neural interactions in disease trajectories (26).

Beyond neurodegeneration, psychiatric disorders also reflect perturbations in this axis. Disruption of microbiota rhythms has been associated with altered neurotransmitter availability, impaired synaptic plasticity, and heightened vulnerability to depression and anxiety (59, 60). For instance, microbiota-derived γ-aminobutyric acid (GABA), dopamine, and serotonin display circadian fluctuations that are crucial for maintaining emotional stability (61). Perturbation of these microbial neurochemicals during circadian misalignment may contribute to mood dysregulation. Social stress models further demonstrate that loss of hierarchical dominance alters gut microbial composition, prefrontal cortex neurotransmission, and circadian-linked behaviors, highlighting the ecological sensitivity of the microbiota–neural axis to psychosocial rhythms (62).

The translational implications of these findings are substantial. Interventions that restore circadian–microbial synchrony, such as time-restricted feeding (TRF), light therapy, and microbiota-targeted therapies, are emerging as promising strategies to stabilize neural function and resilience. For example, temporal dietary interventions not only re-establish microbial rhythmicity but also improve sleep quality and cognitive outcomes (63, 64). Similarly, probiotic supplementation has been shown to enhance sleep efficiency and reduce fatigue, reflecting the ability of microbial modulation to impact circadian-neural outputs (52, 56). More experimental strategies, such as fecal microbiota transplantation from circadian-entrained donors, demonstrate potential in preclinical models for rescuing disrupted corticosterone rhythms and behavioral deficits (7, 57).

Importantly, spaceflight research provides a unique lens into this regulation. Astronauts experience circadian desynchronization due to disrupted light-dark cycles, accompanied by gut microbiota alterations and increased risks of anxiety, depression, and cognitive decline (65–68). These findings parallel terrestrial evidence, reinforcing the universality of the microbiota–circadian–neural interface in regulating psychological resilience under extreme conditions (60). Such insights not only advance human space exploration but also illuminate broader principles of circadian–microbiota–brain interactions applicable to Earth-bound neuropsychiatric health.

In summary, the circadian regulation of the microbiota–neural axis represents a multidimensional and bidirectional framework whereby microbial oscillations and host circadian clocks coalesce to dictate neurophysiological stability. This interface governs neurotransmitter production, stress responsivity, immune signaling, and cognitive function, while its disruption drives vulnerability to neurodegenerative and psychiatric disorders (22, 69, 70). The accumulating evidence across aging, neurodegeneration, psychiatric disease, and space medicine underscores this axis as a central determinant of brain health. Future investigations should delineate the precise molecular circuits and microbially derived metabolites that orchestrate circadian–neural interactions, thereby enabling the development of chrono-microbiome-based therapeutic strategies aimed at re-establishing temporal alignment and preserving brain health across the gut–brain axis.

## Circadian regulation of microbiota–endocrine interactions

The endocrine system is one of the primary mediators of circadian signals to peripheral organs, and its dynamic interaction with the microbiota forms a critical axis orchestrating metabolic, neuroimmune, and reproductive homeostasis. Hormonal secretion across the HPA, hypothalamic–pituitary–gonadal (HPG), and hypothalamic–pituitary–thyroid (HPT) axes is tightly regulated by circadian clocks, with daily oscillations in cortisol, sex steroids, growth hormone, thyroid hormones, insulin, leptin, and ghrelin aligning physiology to external light–dark and feeding cycles (71, 72) (**Figure 1D**). These oscillations not only shape systemic endocrine outputs but also influence microbial composition and activity, given that many microbial taxa possess their own diurnal rhythmicity and are responsive to host hormonal cues. In turn, microbial metabolites such as SCFAs, secondary bile acids, and indoles can act as endocrine-like signals, modulating receptor pathways and altering host hormonal sensitivity (18, 43, 73, 74). This bidirectional regulatory loop underscores a fundamental paradigm: the microbiota–endocrine dialogue is circadian-gated, and disruption of its temporal organization predisposes to metabolic and neoplastic diseases.

At the core of this interaction lies the HPA axis, whose glucocorticoid rhythms exemplify endocrine–microbiome synchrony. Cortisol peaks prior to the onset of activity and exerts pleiotropic roles in resetting peripheral clocks, tuning immune responses, and modulating gut barrier integrity (17, 62, 71). Experimental evidence indicates that microbial communities respond to glucocorticoid fluctuations, with elevated cortisol promoting the expansion of stress-responsive taxa and altering microbial metabolite production, thereby feeding back into host metabolism and inflammatory tone (73, 75). In particular, the gut microbiota regulates the diurnal rhythm of glucocorticoids and that depletion of microbes abolishes rhythmic corticosterone patterns (7). Chronic misalignment of cortisol rhythms, as occurs in shift work, jet lag, or chronic stress, disrupts microbial rhythmicity and increases susceptibility to insulin resistance, obesity, and malignancy (74, 76).

Similarly, the HPT axis illustrates how circadian orchestration of endocrine outputs intersects with microbiota-driven metabolism. Thyroid stimulating hormone (TSH) exhibits pronounced circadian rhythmicity, regulated by the SCN, with downstream oscillations in T3 and T4 coordinating energy expenditure and thermogenesis. Dysregulation of HPT rhythmicity not only perturbs systemic metabolic homeostasis but also alters bile acid metabolism and gut microbial ecology, fostering conditions favorable to metabolic syndrome and even thyroid carcinogenesis (18, 71). In addition, microbial metabolites modulate deiodinase activity and thyroid hormone signaling, providing a mechanistic basis for how microbiota feed back into endocrine regulation (75).

Reproductive endocrinology further highlights the circadian–microbiota–endocrine triad. Gonadal hormones such as estrogen and testosterone follow circadian patterns of secretion, which are critical for reproductive competence and fertility. Estrogens, in particular, interact with the SCN to regulate timing of the luteinizing hormone (LH) surge, demonstrating a tight coupling between circadian clocks and ovulation (72, 77, 78). Importantly, microbiota are emerging as modulators of estrogen availability via the “estrobolome,” a collection of microbial genes encoding β-glucuronidases that influence enterohepatic recycling of estrogens. This microbial modulation of steroid hormones not only impacts reproductive function but also systemic metabolic and oncogenic pathways (76–78). Sex-specific differences in circadian timing and hormonal rhythms have been linked to divergent microbiota profiles, suggesting that biological sex constitutes a key variable in circadian–endocrine–microbial crosstalk (72).

Metabolic hormones such as insulin, leptin, ghrelin, and adiponectin further exemplify circadian–microbial–endocrine interdependence. Insulin sensitivity exhibits robust diurnal variation, with highest glucose tolerance in the morning and lowest in the evening, driven by synchronized SCN and peripheral clock activity. Disruption of these rhythms, whether through altered feeding times or microbiota dysbiosis, leads to impaired glucose homeostasis and insulin resistance, key drivers of type 2 diabetes mellitus (74, 76). Microbial metabolites regulate secretion of incretin hormones, such as GLP-1 and peptide YY, aligning nutrient absorption with circadian phases of feeding (18). In parallel, adipokines such as leptin exhibit circadian oscillations that are both influenced by microbial composition and central to appetite regulation and energy balance (79–81). Ghrelin, secreted in anticipation of meals, is entrained by both feeding schedules and microbial metabolites, creating a tightly woven feedback between circadian clocks, endocrine secretion, and microbial ecology (41, 75, 82).

A striking example of this integration is the pineal hormone melatonin, secreted at night under SCN control. Melatonin not only synchronizes central and peripheral clocks but also directly shapes microbial rhythmicity, with several gut bacterial taxa displaying melatonin-sensitive oscillations in growth and metabolism (83, 84). One classical demonstration is *Enterobacter aerogenes*, which exhibits circadian patterns of swarming motility in response to melatonin signaling (85). Moreover, recent mechanistic work showed that gut microbiota modulate host melatonin production and activate TLR2/4–MyD88–NF-κB signaling to enhance expression of aralkylamine N-acetyltransferase (AANAT), the rate-limiting enzyme in melatonin synthesis (86). Conversely, microbial metabolites such as butyrate can influence pineal melatonin synthesis, reinforcing the reciprocal communication between microbiota and endocrine rhythms (74, 76).

The disruption of circadian–microbiota–endocrine alignment has profound health consequences. Misalignment between feeding times and hormonal rhythms (e.g., insulin, cortisol, melatonin) exacerbates metabolic disorders, while sex-specific circadian disruptions can impair fertility and promote hormone-driven cancers (72, 77). Importantly, epidemiological and experimental studies converge on the notion that circadian disruption, whether through artificial light exposure, irregular sleep, or chronic dietary misalignment, destabilizes endocrine and microbial rhythms simultaneously, creating a feed-forward loop of metabolic dysfunction, immune dysregulation, and oncogenesis (17, 71, 76).

Collectively, the evidence demonstrates that circadian regulation of microbiota–endocrine interactions operates as a central axis of systemic homeostasis. The SCN orchestrates rhythmic endocrine outputs, which entrain microbial oscillations, while microbiota-derived metabolites feed back into endocrine circuits to tune hormonal sensitivity and signaling. The axes most prominently involved, the HPA, HPG, and HPT, illustrate the breadth of this regulation across stress adaptation, reproduction, and metabolism (71, 72, 77). The endocrine factors most relevant such as cortisol, melatonin, thyroid hormones, sex steroids, insulin, leptin, and ghrelin, demonstrate the mechanistic diversity of this crosstalk (73, 74, 82). From a translational standpoint, this triad provides novel therapeutic avenues: chronotherapy to restore hormonal rhythmicity, microbial modulation to stabilize endocrine function, and targeted interventions (e.g., TRF, prebiotics, and probiotics) to reinforce circadian–endocrine alignment (74–76). Thus, circadian regulation of microbiota–endocrine interactions exemplifies a unifying principle of temporal biology: physiological stability and resilience are maintained not by static signals, but by rhythmic synchronization across microbial, endocrine, and circadian networks. Disruption of this triad underlies a spectrum of chronic diseases, while its restoration holds promise for preventive and therapeutic strategies in metabolic, reproductive, and oncologic contexts.

## Circadian regulation of microbiota–immune interactions

Circadian regulation profoundly shapes the dynamic interplay between the microbiota and the immune system, establishing temporal frameworks that optimize host defense, tissue homeostasis, and tolerance. A growing body of evidence indicates that microbial communities exhibit diurnal oscillations in composition, localization, and metabolite production, which in turn entrain or reinforce daily rhythms within host immune cells and mucosal barriers (12, 16, 21, 23, 87) (**Figure 1E**). Conversely, circadian clock genes in immune and epithelial compartments regulate microbial sensing and effector responses, generating a bidirectional crosstalk that tightly links microbiota and immunity across the day-night cycle (21, 88–90). The first demonstration that gut microbiota display diurnal oscillations controlled by host clocks was provided by Thaiss et al., who showed that microbial composition, localization, and function oscillate in synchrony with feeding–fasting and circadian cues (12). Follow-up studies confirmed that these microbial oscillations program host transcriptional rhythmicity and metabolic pathways, revealing that microbiota-derived signals act as transkingdom *zeitgebers* of host circadian physiology (16).

At the mechanistic level, the intestinal microbiota synchronizes innate immune functions to feeding-related circadian cues. Brooks et al. demonstrated that segmented filamentous bacteria (SFB) display rhythmic epithelial attachment, activating group ILC3s and inducing oscillatory STAT3 signaling that drives daily expression of antimicrobial peptides such as REG3γ and lipocalin-2, conferring time-of-day variation in resistance to enteric pathogens (21). This seminal study revealed that microbiota act as temporal cues coordinating epithelial and immune rhythms. Complementary work by Teng F et al. demonstrated that ILC3s themselves possess intrinsic circadian clocks essential for their homeostasis and responsiveness to microbial signals (91). Together, these findings underscore that both microbial and immune clocks co-evolve to sustain rhythmic mucosal defense.

The influence of circadian rhythms on pattern recognition receptor (PRR) signaling is particularly notable. Toll-like receptor 9 (TLR9), responsible for recognizing bacterial and viral DNA, exhibits circadian regulation of both expression and activity. Experimental models demonstrate that responsiveness to TLR9 ligands peaks at specific circadian times, influencing adaptive immune priming during vaccination and altering disease severity during sepsis. These temporal differences underscore that microbial recognition by PRRs is not static but follows circadian cycles shaped by both host clocks and microbial oscillations (89, 92, 93). This rhythmic gating of innate recognition pathways exemplifies how the microbiota–immune axis integrates circadian signals to modulate immune outcomes.

From an ecological perspective, circadian microbial oscillations extend beyond controlled laboratory models into natural populations, where they synchronize with host immune rhythms to influence susceptibility to infection and seasonal disease dynamics. Disruptions to this alignment, whether through light pollution, aging, or environmental stressors, can impair immune rhythmicity, leading to heightened infection risk and dysregulated inflammatory responses (90). These findings indicate that microbiota-driven circadian immunity is not only relevant in controlled conditions but also plays an essential role in shaping host fitness in ecological and evolutionary contexts.

At the cellular and molecular scale, circadian transcriptional–translational feedback loops involving *CLOCK*, *BMAL1*, *PER*, and *CRY* genes and proteins exert pervasive control over immune cell activity. These oscillators regulate leukocyte trafficking, cytokine secretion, and antigen presentation, often in coordination with microbial signals. Wang et al. identified NFIL3 as a circadian transcription factor that integrates microbial and clock-derived cues to control epithelial defense gene expression and systemic metabolism (36). Thus, the microbiota acts as both a *zeitgeber* and a partner to the molecular clock, ensuring immune processes are temporally optimized (1).

Recent clinical and translational studies further demonstrate the immunological consequences of circadian disruption. Zeng et al. recently reported that human peripheral immune subsets, including CD8^+^ central-memory T cells and Treg cells, display strong circadian rhythmicity, accounting for nearly half of their daily variation (94). Other studies showed that inflammatory bowel disease (IBD) patients exhibit suppressed *CLOCK* and *BMAL1* expression in intestinal epithelial cells coupled to dysbiosis and aberrant immune activation (95–97).

Strikingly, recent evidence demonstrates that dietary timing itself modulates inflammatory rhythmicity in a microbiota-dependent manner. In rheumatoid arthritis (RA), meal timing reshapes gut microbial oscillations, influencing diurnal activation of the SIRT5–NF-κB inflammatory axis through *Parabacteroides distasonis* β-glucosidase–mediated release of glycitein, thereby driving time-of-day differences in immune activation (87). This study provides translational proof that chrononutrition and microbiota rhythmicity are tightly intertwined in human inflammatory disease, reinforcing the clinical relevance of circadian–microbiota–immune coupling.

The impact of circadian–microbiota–immune interactions also extends to cancer and immunopathology. Disruption of circadian gene expression alters tumor immune surveillance, promotes immunosuppressive microenvironments, and enhances oncogenesis (98–101). Microbiota-mediated circadian cues, by modulating cytokine profiles and immune checkpoints, may therefore influence not only infection risk but also tumor progression and therapeutic outcomes (94). Beyond tumor initiation, recent findings reveal that circadian disruption can enhance metastatic seeding through microbiota-dependent mechanisms. In a chronic jet-lag model, circadian desynchrony increased gut microbial production of taurocholic acid, which reprogrammed myeloid-derived suppressor cells (MDSCs) toward an immunosuppressive phenotype via H3K4 monomethylation and glycolytic activation, while stabilizing PD-L1 through inhibition of CHIP-mediated ubiquitination. This cascade suppressed CD8^+^ T cell cytotoxicity and accelerated metastatic dissemination of colorectal tumors (28). These results establish that microbial metabolites act as circadian-regulated effectors of metastatic immunity, linking temporal disruption to tumor progression. In this sense, circadian regulation represents a critical dimension of immuno-oncology that warrants deeper exploration. Importantly, microbiota–immune circadian crosstalk is not unidirectional. Recent findings indicate that cytokines and immunoglobulins released during daily immune cycles shape microbial composition and localization, feeding back to reinforce microbial oscillations (36). This bidirectional relationship suggests that perturbations in either immune or microbial rhythms can trigger a vicious cycle of dysregulation leading to systemic pathology (5).

Therapeutically, these insights open avenues for chrono-immunology and microbiome-targeted interventions. TRF, for example, restores intestinal clock gene expression, microbial rhythmicity, and mucosal immune balance in experimental models of colitis, ameliorating inflammation and improving survival (95). Recent studies extend these benefits to neuroimmune contexts, showing that TRF also attenuates AD-related pathology in mouse models by reducing neuroinflammation, enhancing microglial homeostasis, and improving cognitive function (102). Importantly, the protective effect of TRF on AD progression was found to depend on the gut microbiota, as antibiotic-mediated depletion abolished its neuroimmune benefits (103). These findings, together with the RA study, underscore that aligning nutrient intake with circadian and microbial phases can reshape inflammatory trajectories across diseases, highlighting chrono-nutrition as a clinically actionable strategy. Such findings support chrono-nutrition and circadian-aligned probiotic or prebiotic interventions as potential strategies to reinforce immune homeostasis. Similarly, vaccination and immunotherapy may achieve greater efficacy when administered at circadian times that coincide with peak microbial and immune responsiveness (89, 94).

In summary, the circadian regulation of microbiota–immune interactions exemplifies a finely tuned evolutionary adaptation aligning host defense with predictable environmental and microbial challenges. Primary studies now make clear that microbial metabolites, PRR signaling, and immune-cell clocks operate in phase to preserve tissue homeostasis, while circadian misalignment disrupts these axes, driving inflammation and disease. Recent translational findings further show that meal timing can reprogram inflammatory rhythmicity through microbiota-derived metabolites, establishing a direct link between chrono-nutrition and immune resilience. Emerging TRF research further demonstrates that restoring circadian–microbial alignment enhances neuroimmune resilience and mitigates inflammation-associated neurodegeneration (102, 103). Future research must therefore elucidate the molecular mediators of this crosstalk and develop therapeutic strategies that restore circadian–microbial–immune harmony.

## Microbiota-based interventions aligned with circadian rhythms

The growing recognition that circadian rhythms and the gut microbiota engage in a bidirectional dialogue has opened the door to microbiota-based interventions strategically aligned with biological timekeeping (5). Mounting evidence demonstrates that diet, feeding schedules, light exposure, and pharmacological cues not only entrain host circadian clocks but also influence microbial composition and function, while microbial metabolites reciprocally regulate host circadian oscillators at the systemic and cellular levels (104, 105). This reciprocal regulation underlies the emerging concept of “chrono-microbiome medicine,” which seeks to optimize interventions by synchronizing them with circadian biology.

Chrono-nutrition strategies emphasize that the timing of food intake, in conjunction with diet composition, critically shapes diurnal microbial oscillations and thereby modulates host metabolic outputs. Seminal studies have shown that more than half of intestinal microbial taxa undergo time-of-day–dependent fluctuations in abundance and function, with rhythmic peaks in energy harvest, bile-acid metabolism, and SCFA production that reinforce host metabolic rhythmicity (12, 15, 104). Conversely, circadian misalignment, resulting from irregular feeding schedules, late-night eating, or shift-work lifestyles, disrupts these microbial oscillations, dampens peripheral clock activity, and promotes metabolic dysfunction, including obesity, insulin resistance, cardiovascular disease, and cancer susceptibility (14, 105, 106).

A central avenue for intervention is TRF, which restores synchrony between microbial and host clocks by confining caloric intake to biologically optimal windows. Both preclinical and human studies suggest that TRF not only reinstates rhythmicity in microbial communities but also enhances SCFA and bile acid signaling, improving energy homeostasis and attenuating metabolic inflammation (104, 107). In murine models, TRF has been shown to reinforce oscillations of butyrate-producing bacteria and prevent the obesogenic effects of high-fat diets, even when caloric intake remains constant (105). Clinical extrapolation indicates that aligning feeding with circadian phases may be a low-cost, non-invasive intervention to stabilize circadian rhythm disorders, particularly in populations vulnerable to chrono-disruption such as shift workers and individuals with metabolic syndrome (106, 108).

Probiotics and prebiotics further represent promising microbiota-targeted interventions with circadian applications. Specific strains of *Lactobacillus* and *Bifidobacterium* display rhythmic fluctuations in colonization density and metabolite secretion, suggesting that timed administration could maximize efficacy (109, 110). Probiotics not only reinforce the rhythmic production of SCFAs and indoles but also modulate systemic inflammation and neurotransmitter availability, thereby supporting metabolic, neuroimmune, and sleep-wake regulation (110, 111). Importantly, probiotic supplementation has been associated with improved glucose tolerance, enhanced sleep quality, and reduced anxiety-like behavior, outcomes tightly connected to circadian alignment of the gut-brain axis (109, 111). Prebiotics, such as dietary fibers and polyphenols, similarly modulate circadian microbial rhythms by promoting the diurnal expansion of butyrate producing taxa, which regulate clock gene expression in hepatocytes and modulate appetite-related enteroendocrine signaling (105, 106).

In addition to nutritional interventions, pharmacological chronotherapy represents a powerful approach to exploit circadian-microbiota interactions. Chronopharmacology has revealed that drug absorption, metabolism, and efficacy vary according to circadian phases, and the microbiota is increasingly recognized as a mediator of these time-dependent effects. For instance, microbial β-glucuronidases modulate the toxicity of chemotherapeutics such as irinotecan, and their activity follows circadian oscillations, implying that timed probiotic or enzymatic inhibition could reduce side effects (112–114). Moreover, microbial regulation of bile acids and tryptophan metabolites influences hepatic and neural clock genes, suggesting that microbiota-directed therapies could serve as adjuncts to conventional chronotherapy (23, 115, 116). Importantly, integrating microbiota-based strategies with personalized chronotherapy may advance precision medicine, especially in metabolic, cardiovascular, oncological, and neuropsychiatric contexts (110, 117).

Another novel field of exploration is chrono-microbiota modulation (CMM), an integrated strategy that combines dietary timing, probiotics, and circadian-targeting drugs. CMM aims to reestablish synchrony between host and microbial clocks, thereby improving outcomes in conditions where circadian misalignment exacerbates pathology. For example, in cardiovascular disease, rhythmic microbial metabolites such as SCFAs and trimethylamine-N-oxide (TMAO) affect endothelial function and plaque stability; aligning microbiota-targeted therapies with circadian windows may mitigate early-morning peaks in cardiovascular events (108). Similarly, in sleep disorders, microbial metabolites including acetate and butyrate influence the expression of *PER* and *BMAL1* genes in hepatocytes and the brain, suggesting that microbiota-based interventions could stabilize circadian oscillators and improve sleep–wake cycles (106, 109). Recent multi-omics clinical studies confirm that circadian rhythm disorders are accompanied by specific microbial and metabolite alterations, including *Lachnospiraceae* and bile acid derivatives, which can be leveraged as biomarkers for tailored microbiota-based therapy (107).

Beyond dietary and pharmacological strategies, antibiotic exposure has emerged as a potent disruptor of microbial and host circadian organization. Broad-spectrum antibiotics abolish diurnal oscillations in microbial composition and metabolite production, leading to the loss of rhythmic short-chain fatty acids, bile acids, and tryptophan derivatives that normally entrain peripheral clocks (118–120). This circadian collapse extends to the host, impairing rhythmic gene expression in hepatic and intestinal tissues, perturbing glucose and lipid metabolism, and altering corticosterone and sleep–wake rhythms (121). Moreover, antibiotic-induced microbiota depletion attenuates rhythmic immune surveillance in mucosal and systemic compartments, heightening susceptibility to inflammation and infection (21). Collectively, these findings highlight the need to consider timing, duration, and microbial restoration strategies in antibiotic therapy to preserve circadian and metabolic homeostasis.

Despite these advances, significant challenges remain. First, there is marked interindividual variability in both circadian profiles and microbial ecology, making personalized interventions essential (106, 111). Wearable devices, digital circadian profiling, and salivary omics now enable real-time tracking of circadian biomarkers, allowing alignment of interventions with each individual’s chronotype (117). Second, clinical translation requires rigorous trials to determine the optimal strains, prebiotics, or feeding schedules for specific populations. Furthermore, safety and ecological risk assessments are needed, as interventions altering microbial rhythms could inadvertently disrupt other host–microbe interactions (108, 111). Finally, integration of artificial intelligence with multi-omics datasets will be critical to identify predictive biomarkers, optimize therapeutic timing, and reduce intersubject variability (107, 117).

Ultimately, microbiota-based interventions aligned with daily rhythms represent a rapidly advancing frontier at the nexus of chronobiology, nutrition, and systems medicine. By harmonizing microbial and host clocks through TRF, temporally targeted probiotics and prebiotics, and integrated chronotherapy, these strategies provide innovative avenues to reestablish metabolic, neuroimmune, and endocrine homeostasis. The overarching objective is to transform mechanistic discoveries into clinically actionable chrono-microbiome interventions capable of preventing or mitigating circadian-related disorders, including metabolic syndrome, cardiovascular disease, sleep disturbances, and cancer.

## Conclusions, limitations, and future perspectives

The cumulative evidence from molecular, translational, and clinical studies underscores that circadian rhythms and the microbiota are deeply intertwined regulators of systemic physiology, spanning metabolic, immune, neuroendocrine, and behavioral domains. Disruption of this delicate temporal balance, whether through genetic alterations, lifestyle factors such as shift work, obesogenic diets, or exposure to artificial light, drives systemic misalignment that accelerates metabolic dysfunction, immune dysregulation, neuropsychiatric vulnerability, and endocrine imbalance (17, 33, 105). The growing appreciation of the microbiota as both a mediator and modulator of daily rhythms places it at the core of a temporal systems biology framework with profound therapeutic implications.

From a metabolic standpoint, circadian regulation of feeding-fasting cycles, nutrient absorption, and energy expenditure is tightly orchestrated by microbial oscillations. Time-restricted eating and other chrono-nutrition strategies restore alignment of host and microbial clocks, improving glucose and lipid metabolism while counteracting diet-induced obesity and diabetes (33, 105). Importantly, microbiota-derived metabolites such as SCFAs exhibit rhythmicity, serving as both energy substrates and signaling molecules that reinforce circadian synchronization of peripheral tissues (33, 73). Future research should expand omics-driven approaches to unravel tissue-specific microbiome-circadian signatures that could serve as predictive biomarkers for metabolic health trajectories.

The endocrine system emerges as a pivotal mediator in this bidirectional dialogue. Hormones such as melatonin, cortisol, thyroid hormones, and glucocorticoids oscillate under circadian control and, in turn, entrain peripheral clocks while influencing microbial composition and activity (2, 17, 57). Recent insights into microbial contributions to melatonin metabolism suggest that the gut microbiota may represent a novel therapeutic target for restoring circadian rhythmicity and treating rhythm-related disorders (19). This axis highlights a promising translational window for chronotherapeutic interventions that combine pharmacological, dietary, and microbial strategies to realign disrupted hormonal and microbial rhythms.

In the neuroimmune context, microbial oscillations modulate brain function through immune, neural, and neuroendocrine pathways. The gut-brain axis is strongly influenced by microbial metabolites, neurotransmitter precursors, and vagal signaling, which oscillate diurnally and impact stress resilience, sleep-wake cycles, and mood regulation. Circadian misalignment has been associated with heightened neuroinflammation, cognitive decline, and psychiatric disorders, mediated in part by microbial dysbiosis (88). Furthermore, circadian-microbiota-immune crosstalk regulates intestinal barrier integrity and systemic immune tone, with disruptions predisposing to inflammatory and autoimmune diseases (55, 95). These insights call for integrative investigations into how timed microbiota interventions may mitigate neuroimmune pathologies, particularly in disorders such as depression, multiple sclerosis, and AD.

Immune regulation represents another critical frontier in circadian-microbiota interactions. The microbiome entrains host immune rhythms, modulating recruitment, trafficking, and effector function of immune cells through SCFAs, tryptophan metabolites, and bacterial ligands that oscillate daily (55, 95). Disrupted microbial rhythms impair mucosal defense and promote chronic inflammation, with downstream consequences for cancer susceptibility and autoimmune disease progression (73). Future perspectives should include longitudinal human studies to determine whether restoring microbial rhythmicity can improve outcomes in immune-mediated disorders and optimize responses to immunotherapy (122).

Despite major advances, significant limitations constrain current understanding of circadian–microbiota crosstalk in humans. Most mechanistic evidence derives from murine models with targeted clock gene deletions, whereas human data on naturally occurring circadian gene variants and their impact on microbiota function are scarce. Human studies are further limited by low temporal resolution, as microbiome samples are often collected at single or sparse time points, obscuring diurnal dynamics in microbial localization and metabolite flux. Moreover, distinguishing endogenous circadian rhythms from feeding- or behavior-driven daily fluctuations remains challenging outside controlled laboratory settings. Additional confounders, including diet composition and timing, light exposure, medications, sleep duration, and stress, further complicate causal interpretation. Methodological heterogeneity, encompassing sequencing depth, analytical pipelines, and reliance on stool rather than mucosal samples, undermines reproducibility across studies. Finally, interindividual variability in chronotype, sex, age, geography, and lifestyle introduces high biological noise, highlighting the urgent need for standardized, longitudinal, multi-omic, and multi-ethnic studies to establish causal links and enable translational chrono-microbiome medicine.

Looking ahead, the integration of multi-omic technologies, metagenomics, metabolomics, transcriptomics, and epigenomics, will be essential for constructing high-resolution temporal maps of microbiome-host interactions. These tools will enable the identification of microbial signatures predictive of circadian alignment or misalignment, facilitating precision diagnostics and early intervention strategies (33, 73). Moreover, personalized circadian medicine, incorporating wearable technologies and digital chronotyping, will likely converge with microbiome science to deliver individualized therapeutic regimens (2, 57).

Therapeutically, the emerging paradigm of circadian precision medicine envisions the use of timed microbiota-targeted interventions, such as probiotics, prebiotics, dietary restriction, fecal microbiota transplantation, and chronopharmacology, to re-establish temporal harmony across organ systems. In particular, aligning drug administration with circadian and microbial cycles holds promise for enhancing efficacy while minimizing toxicity in metabolic, endocrine, and neuroimmune disorders (2, 57, 105). Furthermore, targeting microbial contributions to endocrine mediators such as melatonin and leveraging diet-driven modulation of microbial rhythms may represent the next generation of chrono-microbiome interventions (19).

Nonetheless, substantial gaps remain. Much of the current evidence derives from animal models, and translation to human physiology requires robust, longitudinal, and multi-ethnic clinical trials (95, 105). Another challenge lies in disentangling causality from correlation: while circadian disruption and microbial dysbiosis are strongly associated with disease, establishing direct mechanistic links is essential for clinical translation. Moreover, methodological heterogeneity in microbiome research, including sequencing depth, sampling frequency, and the use of 16S rRNA versus shotgun metagenomics, continues to limit reproducibility and comparability across studies (55, 73). Standardization of analytical frameworks will therefore be critical moving forward.

In conclusion, circadian modulation of microbiota-mediated crosstalk represents a rapidly expanding frontier at the intersection of chronobiology, systems medicine, and microbial ecology. By embracing multi-omic integration, advancing chronotherapeutic strategies, and conducting rigorously designed clinical trials, future research can unlock the full therapeutic potential of this axis. The ultimate goal is to translate mechanistic insights into clinically actionable interventions that restore temporal harmony, enhance resilience against metabolic, neuroimmune, and endocrine disorders, and pave the way for personalized chrono-microbiome medicine.
